# Cyclodextrin-Assisted Extraction Method as a Green Alternative to Increase the Isoflavone Yield from *Trifolium pratensis* L. Extract

**DOI:** 10.3390/pharmaceutics13050620

**Published:** 2021-04-26

**Authors:** Jurga Andreja Kazlauskaite, Liudas Ivanauskas, Jurga Bernatoniene

**Affiliations:** 1 Department of Drug Technology and Social Pharmacy, Lithuanian University of Health Sciences, LT-50161 Kaunas, Lithuania; jurga.andreja.kazlauskaite@stud.lsmu.lt; 2 Institute of Pharmaceutical Technologies, Lithuanian University of Health Sciences, LT-50161 Kaunas, Lithuania; 3 Department of Analytical and Toxicological Chemistry, Lithuanian University of Health Sciences, LT-50161 Kaunas, Lithuania; liudas.ivanauskas@lsmuni.lt

**Keywords:** innovative strategies, *Trifolium pratensis* L., red clover, isoflavones, aglycones, excipients, cyclodextrins, extractions

## Abstract

*Trifolium pratense* L. is receiving increasing attention due to the isoflavones it contains, which have been studied for their benefits to human health. A common problem with isoflavone aglycones is a rather low water solubility and limited pharmaceutical applications. The use of excipients, such as cyclodextrins in the production of isoflavone rich extracts, could become one of the new strategies for the extraction of target compounds. The aim of this study was to evaluate an eco-friendly method using the effects of *α*-, *β*- and *γ*-cyclodextrins for isoflavone solubilization in plant extracts in comparison to a standard extract without excipients. Extractions of red clover were prepared using ultrasound-assisted combined with thermal hydrolysis and heat reflux. It was determined that cyclodextrins significantly increased the isoflavones aglycone yields. By increasing cyclodextrins in the extraction media from 1 to 5%, the daidzin concentration increased on average by 1.06 (*α*-cyclodextrins), 1.4 (*β*-cyclodextrins) and 1.25 (*γ*-cyclodextrins) times. Genistein concentration increased using *α*- and *γ*-cyclodextrins (1.28 and 1.12 times, *α*- and *γ*-cyclodextrins, respectively), but decreased using *β*-cyclodextrins. The results showed that the cyclodextrin-assisted extraction enhanced the yields of isoflavones from red clover, which suggests using cyclodextrins as a green alternative and a cost-effective method to increase its pharmaceutical application.

## 1. Introduction

**Figure 1:**
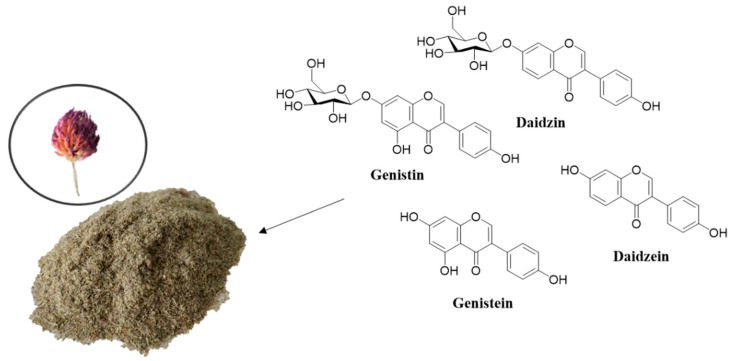
Milled red clover flower buds (0.5 mm) and the main isoflavones found in its extracts.

*Trifolium pratense* L. (red clover) (Figure 1) is a member of the family Leguminosae or Fabaceae. It is a short-lived biennial plant which has been used for its wide health benefits, which come from isoflavones [1]. The main isoflavones found in red clover are daidzein, genistein, formononetin, and biochanin A, as well as their glycosides (such as genistin, daidzin) [2,3].

Isoflavones are part of a large family of secondary plant metabolites called flavonoids [4]. Daidzin and genistein are the aglycones belonging to the phytoestrogen class and possessing a broad range of pharmacological properties that reduce tumors, menopausal symptoms, osteoporosis, and signs of aging. Isoflavones have also been reported to improve learning skills and memory in menopausal women and to aid in the prevention and treatment of heart disease and diabetes [5,6,7]. Phytoestrogens structurally resemble naturally occurring estrogens in women and compete with them for binding estrogen receptors. These compounds have a stronger affinity for binding ER-*β* than ER-*α*. By interacting with ERs, isoflavones can exert estrogenic effects in humans. However, depending on the dose or the duration of exposure, isoflavones can also act as antiestrogenic agents [8]. They are currently heralded as offering potential alternative therapies for a range of hormone-dependent conditions, including cancer, menopausal symptoms, and cardiovascular disease [9,10].

In plants, the most widely studied isoflavones, genistein and daidzein, are usually encountered as conjugates and are hydrolyzed to aglycones (biologically active form) in the human gut [11]. The isoflavone aglycones are absorbed faster and in greater amounts than their glucosides. Therefore, hydrolysis of the sugar moiety is an essential prerequisite for the bioavailability of isoflavones [12].

The estrogenic and antioxidant activities of daidzein and genistein, as mentioned before, are conditioned to the aglycone form, which presents reduced solubility in aqueous media [13,14]. Therefore, to preserve bioactive molecules and improve certain properties, they need to be used with finishing formulation with the capacity to improve needed characteristics. Additional materials, such as excipients, may be used to extract isoflavone aglycones in water or another solvent and thus increase their yield in extract. Cyclodextrins (CDs) can be suitable compounds for increasing the solubility of isoflavones. CDs can interact with appropriately sized molecules to result in the formation of inclusion complexes. These noncovalent complexes offer a variety of physicochemical advantages compared to the unmanipulated drugs, including the possibility for increased water solubility and solution stability [15].

There has been an increasing demand in recent years for cheaper, safer, and more eco-friendly alternatives to organic solvents. CD-based extraction is an emerging “green” technology of great potential [16]. CDs are being explored in the extraction of phenolic compounds, such as phenolic acids, flavonoids and stilbenes from various types of natural sources. The green extraction of phenolic compounds using CDs provides the possibility for a more effective exploitation of natural plant resources and wider use of phenolic compounds in the food and nutraceutical industries [17].

**Figure 2:**
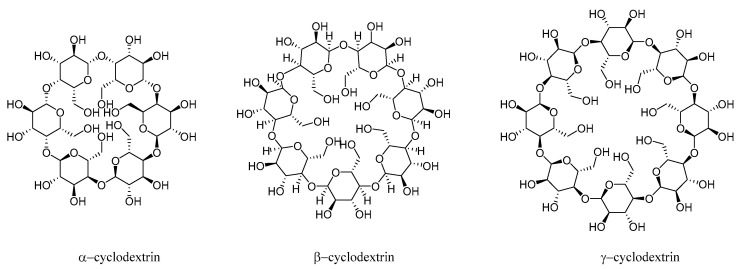
Chemical structures of *α*-, *β*- and *γ*-CD.

CDs are cyclic oligosaccharides composed of 6, 7, or 8 glucose units, named *α*-, *β*-, or *γ*-cyclodextrin, respectively (Figure 2) [18]. They have a rigid conical molecular structure with a hydrophobic interior and hydrophilic exterior. The internal cavity of these molecules can include a wide range of guest molecules, ranging from polar compounds, such as alcohols, acids, amines, and small inorganic anions, to non-polar compounds, such as aliphatic and aromatic hydrocarbons, while the hydrophilic exterior helps CDs to interact favorably with water [19].

The molecular interaction of isoflavones (genistein and daidzein) and *β*-CD and its derivatives was studied by Shujing Li et al. [20] and V. Crupi et al. [21]. In these studies, it was concluded that CDs form host–guest complexes and improve the solubility of pure daidzein and genistein in water due to the formation of an inclusion complex [20,21]. Another published study investigated the formation of complexes of native cyclodextrins and pure genistein. Genistein was found to form the lowest number of complexes with *α*- CD. With *β*- and *γ*-CDs, genistein formed complexes, but at different rates. The resulting complexes were water soluble [22]. CDs can form dynamic solid inclusion complexes with a wide variety of solid, liquid, and gaseous compounds by the process of molecular complexation. There is no breakage or formation of covalent bonds during the formation of an inclusion complex [23]. The driving forces leading to the inclusion complex formation include the release of enthalpy-rich water molecules from the cavity, electrostatic interaction, Van der Waals interaction, hydrophobic interaction, hydrogen bonding, release of conformational strain, and charge–transfer interaction [24].

Choosing the right type of CD is important, since this factor can influence the extraction yield and the selection of compounds from the sample matrix. The diameter and volume of CDs increase with the increasing number of glucose units. Therefore, *β*-CD is widely used due in its appropriate cavity size; *γ*-CD is more suitable for moderate or larger sized compounds, while *α*-CD is limited to accommodate some small guest molecules [17].

To our knowledge, this is the first study using *α*-, *β*- and *γ*-CDs in *Trifolium pratensis* L. extracts to increase aglycones daidzein and genistein yield using water and 50% ethanol as a solvent. Thus, the present study was undertaken in order to evaluate the eco-friendly method of using the effects of *α*-, *β*-, and *γ*-CDs for isoflavone solubilization in red clover extracts in comparison to a standard extract without excipients.

## 2. Materials and Methods

### 2.1. Materials

Red clover samples were collected in red clover fields in Laičiai, Kupiškis district, Lithuania (latitude 55°53′24.2″ N; longitude 25°19′36.0″ E). The collections of flower buds and flowers were made on the 26th of September. Samples were dried and stored at room temperature. Before use, clover flowers were ground to a fine powder using Ultra Centrifugal Mill ZM 200 (Retsch, Haan, Germany). Grinding was performed at 4025 g using a 0.5 mm trapezoid hole sieve.

HPLC grade and analytical-grade reagents were used: standards of genistein, genistin, daidzein and daidzin (Sigma Aldrich, Steinheim, Germany); hydrochloric acid, sodium hydroxide, acetic acid, methanol, acetonitrile, *α*-, *β*- and *γ*-CDs (Sigma Aldrich, Hamburg, Germany); ethanol (96%) (Vilniaus Degtine, Vilniaus, Lithuania); purified water was prepared with GFL2004 (GFL, Burgwedelis, Germany). Deionized water was prepared with Milipore, SimPak 1 (Merck, Darmstadt, Germany).

### 2.2. Preparation and Extraction of Plant Material

#### 2.2.1. Plant Material Moisture Determination

The moisture content of the milled red clover flowers was determined using a KERN MLB apparatus (KERN & Sohn GmbH, Balingen, Germany). Aa amount of 0.3 ± 0.01 g of the material was placed in the apparatus and heated to 105 °C. At the end of the operation, the device provided the moisture content of the material. The moisture of the plant material varied from 7 to 7.4%.

#### 2.2.2. Ultrasound-Assisted Extraction with Thermal Hydrolysis (UAE)

Ultrasound-assisted extraction was performed using an ultrasound bath (frequency 38 kHz) (Grant Instruments™ XUB12 Digital, Cambridge, England). A sample of 0.3 ± 0.001 g of dried and milled flower heads was macerated in 10 mL of solvent. The extraction of isoflavones was performed by employing different extraction conditions: solvent (50% *v/v* ethanol and purified water) and extraction time (10 or 30 min), with the processing temperature of 40 ± 2 °C (the temperature is regulated automatically by the ultrasonic bath) [25,26].

Thermal hydrolysis was completed by transferring the extract to a 250 mL round bottom flask. It was refluxed in a sand bath at 100 °C for 1 h. After the procedure, the mixture was left to cool down and then centrifuged with Sigma 3-18K centrifuge (Sigma, Osterode am Harz, Germany) for 10 min at 3382× *g*, followed by the decantation of the supernatant. The extracts were filtered through PVDF syringe filters (pore size 0.22 μm, Frisenette, Knebel, Denmark) prior to HPLC analysis. Sample preparation conditions are listed in Table 1.

#### 2.2.3. Heat-Reflux Extraction (HRE)

Amounts of 0.3 ± 0.001 g of dried and milled flower heads were mixed with 10 mL of solvent (50% ethanol *v/v* or purified water) in a 250 mL round bottom flask and refluxed in a sand bath at 100 °C for 1 h. After that, the mixture was left to cool at a temperature of 25 ± 2 °C. The samples were centrifuged for 10 min at 3382× *g*, followed by the decantation of the supernatant. The extracts were filtered through PVDF syringe filters (pore size 0.22 μm) prior to HPLC analysis. Sample preparation conditions are listed in Table 1.

### 2.3. The Use of CDs in the Preparation of Extracts

Samples were modified with *α*-, *β*- or *γ*-CDs. The extracts were made under the same conditions as previously listed. Purified water or 50% of ethanol (*v*/*v*) was used as the solvent, and the excipient was added to the extraction mixture. The same amount of 0.1 ± 0.001 g of *α*-, *β*- or *γ*-CDs was added to the extraction mixture samples (10 mL) to prepare samples with CD concentrations of 1% (*w*/*v*). Another amount of 0.5 ± 0.001 g of *α*-, *β*- or *γ*-CDs was added to the extraction mixture samples (10 mL) to prepare samples with CD concentrations of 5% (*w*/*v*). The excipient amount was based on the quantity of solvent in the extract. The samples were centrifuged for 10 min at 3382× *g*, followed by the decantation of the supernatant. The extracts were filtered through PVDF syringe filters (pore size 0.22 μm) prior to HPLC analysis. Sample preparation conditions are listed in Table 1.

### 2.4. HPLC–PDA Conditions

**Figure 3:**
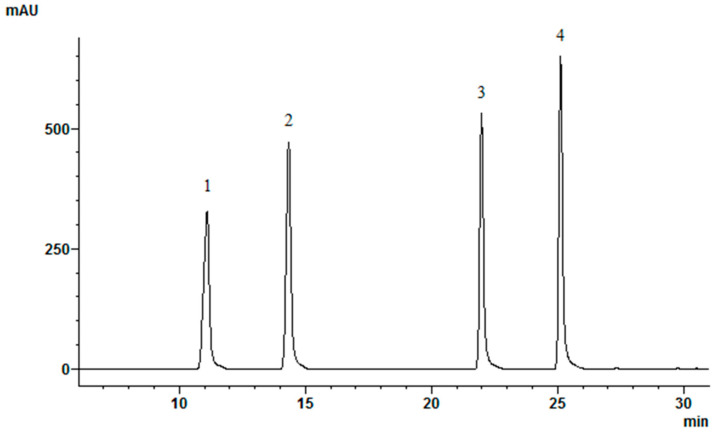
Isoflavone separation HPLC-DAD chromatogram: 1-daidzin; 2-genistin; 3-daidzein; 4-genistein.

HPLC analyses were carried out using the Shimadzu Nexera X2 LC-30AD HPLC system (Shimadzu, Tokyo, Japan), consisting of a quaternary pump, an on-line degasser, a column temperature controller, the SIL-30AC autosampler (Shimadzu, Tokyo, Japan), equipped with the CTO-20AC thermostat (Shimadzu, Tokyo, Japan) as well as the SPD-M20A diode array detector (DAD). For the determination of polyphenols, an ACE 5 C18 250 × 4.6 mm column (Advanced Chromatography Technologies, Aberdeen, Scotland) was used. The mobile phase consisted of solvent A (acetic acid/methanol/deionized water) (1:10:89 *v*/*v*/*v*) and solvent B (acetic acid/methanol) (1:99 *v*/*v*/*v*). The linear gradient elution profile was as follows: 80% A/20% B at 0 min; 30% A/70% B at 30 min; 90% A/10% B at 39 to 40 min. The flow rate was 1 mL/min, and the injection volume was 10 μL. Absorption was measured at 260 nm. Quantification of isoflavone compounds was performed using reference standards of daidzein, genistein, daidzin and genistin. The linear calibration curves were constructed (daidzein R^2^ = 0.9999; genistein R^2^ = 0.9999; daidzin R^2^ = 0.9999; genistin R^2^ = 0.9999); the peak areas were used for quantification. Linear calibration curve functions were as follows: f(x) = 59664.2x + 37164.6 (daidzein), f(x) = 73083.1x + 44202.9 (genistein), f(x) = 38202.1x + 19377.4 (daidzin), f(x) = 49602.9x + 24083.3 (genistin). The contents were expressed as μg/g dry weight (dw). The range of linearity of daidzein: 0.43–221 µg/mL; genistein: 0.43–218 µg/mL; daidzin: 0.32–165 µg/mL; genistin: 0.3–151.5 µg/mL. Isoflavone separation HPLC-DAD chromatogram is provided in Figure 3.

#### 2.4.1. Recovery Test of Isoflavones

The accuracy of HPLC-DAD was investigated using a modified Grazina et al. recovery test method [27]. Two selected ethanolic red clover extracts were prepared as described in Section 2.2.2. The UA sample was prepared using only ultrasound (processing time—10 min, at temperature of 40 ± 2 °C). The UAH sample was prepared using ultrasound for 10 min (at temperature of 40 ± 2 °C) and then heating the sample under reflux for 1 h. The samples were centrifuged for 10 min at 3382× *g*, followed by the decantation of the supernatant. The extracts were filtered through PVDF syringe filters (pore size: 0.22 μm) prior to HPLC analysis. Each sample was injected 6 times (*n* = 6). The results of isoflavones recoveries are provided in Table 2.

#### 2.4.2. Isoflavones Obtained via CD-Assisted Extraction Release Analysis with HPLC-DAD

The isoflavone release from the CD complex was investigated using pure isoflavone standards (genistein, daidzein, genistin and daidzin). Test samples were prepared using 1 mL of isoflavones standards mixed with 9 mL of 50% *v/v* ethanol or purified water with 0.1 ± 0.001 g of *α*-, *β*- or *γ*-CD. The samples were sonicated for 10 min (at temperature of 40 ± 2 °C) and after that heated under reflux for 1 h. The samples were filtered through PVDF syringe filters (pore size 0.22 μm) prior to HPLC analysis. The results are shown in Table 3.

### 2.5. Statistical Analysis

Data are presented as mean ± standard deviation (SD). All experiments were performed in triplicate. Statistical analysis of the results was performed with SPSS 20.0 (IBM Corporation, Armonk, NY, USA). One-way ANOVA was used to investigate the differences between extractions. Post hoc comparisons of the means were performed according to Tukey’s HSD test. The means of compared samples were considered significantly different when *p* < 0.05.

## 3. Results and Discussion

### 3.1. Quantification of the Aglycones Using Purified Water and CDs as an Excipient

Before the experiment optimal conditions were selected for this research, different concentrations of ethanol and two temperatures of ultrasound were selected in order to obtain higher amounts of isoflavones. By extending the ultrasound processing time from 10 to 30 min, daidzein yields decreased, but genistein using 50% of ethanol increased (EU1(KE4) and EU2(KE5) samples) (Table 4). By increasing ethanol concentrations and the ultrasound processing temperature, isoflavone yield decreased. This is why in this study, the ultrasound temperature was 40 °C (processing time 10–30 min, later used ethanol concentration 50%).

The yield of isoflavones was determined in aqueous extracts obtained from dried and milled Trifolium pratensis L. flower heads material by UAE and HRE. Isoflavones were determined using HPLC-PDA. To make sure that all the isoflavones obtained via CD-assisted extraction were released from the complex during HPLC-DAD analysis, we conducted an additional experiment (Section 2.4.2). The results showed (Table 2) that we obtained the same amounts of isoflavones as we had added. Therefore, all the isoflavones in the extracts, determined by HPLC-DAD analysis, were separated from the complexes with CDs, and there were no isoflavones still left in the complexes.

The use of HRE and UAE methods allows high yields of isoflavones to be obtained. UAE waves are transmitted through the liquid medium, damaging the plant wall, resulting in an improved solvent penetration. Therefore, bioactive components can be extracted in minutes [28]. The presence of heat during isoflavone extractions triggers chemical changes, with the decarboxylation of malonyl-glucosides to acetyl-glucosides and the breakdown of the ester bond being frequently observed, the latter leading to the formation of glucosides [26]. When using high temperatures, glycosides could convert to aglycones, if the pH is right for the hydrolysis. However, the temperature and processing time should be closely monitored and properly selected, because genistein and daidzein could degrade at high temperatures [29]. Due to their simplicity and efficiency, HRE and UAE with thermal hydrolysis have been used in the extraction of isoflavones in this study.

**Figure 4:**
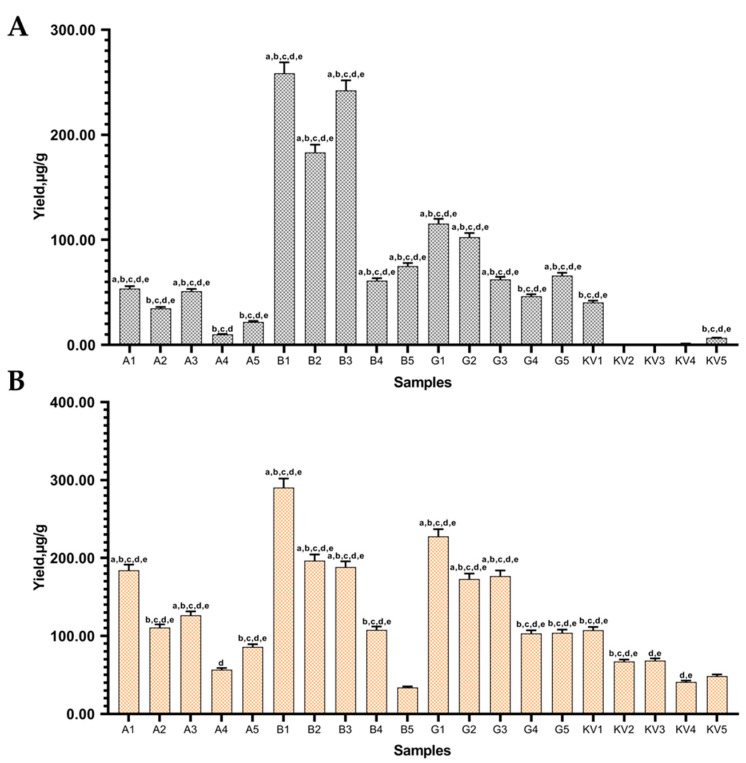
Quantitative yield of (**A**) genistein and (**B**) daidzein using excipients (1%). Control samples without excipients (KV1-5), samples with *α*-CD (A1-5), *β*-CD (B1-5), *γ*-CD (G1-5). ^a^ *p* < 0.05 vs. KV1, ^b^ *p* < 0.05 vs. KV2, ^c^ *p* < 0.05 vs. KV3, ^d^ *p* < 0.05 vs. KV4, ^e^ *p* < 0.05 vs. KV5. Sample codes are provided in Table 1.

The use of CDs in an aqueous solution as extraction media can be considered a green extraction since water is the main solvent and the existence of CD hydrophobic cavity boosts the extraction of phenolic compounds, including isoflavones, due to the formation of the inclusion complex [17]. Comparing control (KV1-5) samples with samples prepared using CDs, it was determined that excipients enhanced aglycone yields (Figure 4).

The highest aglycone yields were detected in sample B1 (258.67 ± 10.34 genistein and 290.33 ± 11.61 µg/g daidzein); the results were statistically significant compared with control samples. It was observed that using *α*- and *β*-CDs (A2-5 and B2-5 samples using *α*- and *β*-CDs, respectively) during UAE and extending the sonication time from 10 to 30 min resulted in increased aglycone yields. However, using *γ*-CDs (G2-3 samples) during UAE (with additional thermal hydrolysis) prolonged the processing time and reduced aglycone amounts in extracts (Figure 4A,B). Almost all of the samples (excluding A2, A3, A4 and B5 samples) showed higher extraction results (*p* < 0.05) than the control samples without excipients, and the use of the HRE method produced higher isoflavones extraction results than UAE (Figure 4A,B). Using only UAE, without thermal hydrolysis (samples A, B; G 4-5), showed poor (Figure 4A,B) but statistically significant results (compared with controls) in extracting daidzein (Figure 4A). To obtain higher yields of daidzein and genistein, thermal hydrolysis is a necessary step.

The increased yield of isoflavones in extracts with excipients can be explained by the structure of CDs. The CD encapsulation of active compounds changes the compounds’ physicochemical properties, such as their aqueous solubility and chemical stability. The CD molecule forms a hydrophilic shield around the appropriate lipophilic moiety of the drug molecule. This increases the apparent aqueous solubility of the active compounds. The CD can also protect chemically labile drug molecules from potentially corrosive environments and, in this way, reduce or even prevent drug hydrolysis, oxidation, racemization and enzymatic decomposition [17,30]. A. The studies of Daruházi et al. focused on the pure genistein and native, chemically non-modified CDs [22]. Their findings were similar to those obtained in this study: the interaction between genistein and *α*-CD with the smallest cavity size was found to be negligible compared to *β*- and *γ*-CDs. Therefore, genistein has the lowest affinity and cannot form a stable complex with *α*-CD. However, in this study, the results obtained with *α*-CD were statistically significant compared to the control samples (KV1-5) (Figure 4).

Additionally, the target compounds (in the case of this study, isoflavones) must be hydrophobic to form a complex with CDs due to the hydrophobic cavity of the CD. The more hydrophobic the guest is, the stronger the interaction is between the host and guest. Genistein and daidzein have poor water solubility, but daidzein is more lipophilic than genistein [31,32]. However, glycosides are naturally polar due to the availability of at least one sugar moiety in the molecular structure [33]. This physical property of isoflavones explains why the aglycone yield in the extracts with CDs increased and no glycosides were found.

### 3.2. Influence of Cyclodextrin Increase on Aglycone Yield

**Figure 5:**
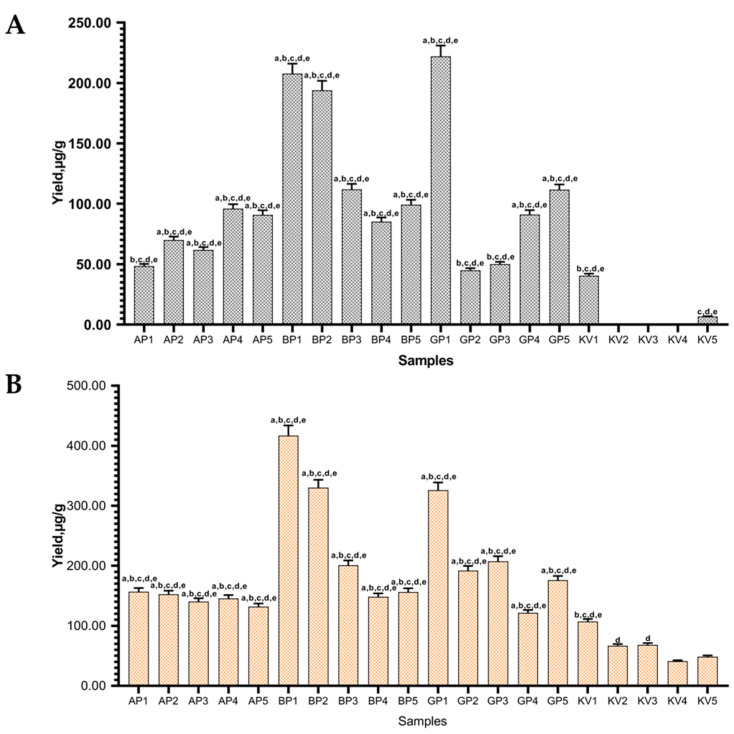
Quantitative yield of (**A**) genistein and (**B**) daidzein using excipients (5%). Control samples without excipients (KV1-5), samples with *α*-CD (AP1-5), *β*-CD (BP1-5), *γ*-CD (GP1-5). ^a^ *p* < 0.05 vs. KV1, ^b^ *p* < 0.05 vs. KV2, ^c^ *p* < 0.05 vs. KV3, ^d^ *p* < 0.05 vs. KV4, ^e^ *p* < 0.05 vs. KV5. Sample codes are provided in Table 1.

To enhance isoflavone yields, CD concentrations were increased to 5%. The highest (*p* < 0.05) amounts of isoflavones were obtained from BP1 and GP1 samples, which were prepared using *β*-CDs and *γ*-CDs, respectively (Figure 5). Sample BP1 yielded the highest amount of daidzein (417.1 ± 16.68 µg/g) and GP1 genistein (222.133 ± 8.82 µg/g).

However, the increase in CDs increased the amount of daidzein more than genistein. Daidzein yields increased on average by 1.06 (*α*-CDs), 1.4 (*β*-CDs) and 1.25 (*γ*-CDs) times when the CDs were increased from 1 to 5%. Genistein yields increased using *α*- and *γ*-CDs (1.28 and 1.12 times, *α*- and *γ*-CDs, respectively), but decreased using *β*-CD. Nevertheless, as in extracts with 1% CDs, the glycosides, daidzin and genistin were not detected in the extracts with 5% of excipients.

As previously mentioned, CD forms complexes with hydrophobic compounds. According to lipophilicity, expressed as the logarithm of the octanol–water partition coefficient log P o/w, genistein has 2.04 and daidzein 2.24, which means, that daidzein is more hydrophobic than genistein and as a result can form host–guest complexes with the CDs more actively [34]. The increase in the daidzein yield indicates that in high temperatures it can form more stable complexes with *β*-CDs and *γ*-CDs compared with genistein.

In this study, a temperature of up to 100 °C was employed. In the literature, phase-solubility studies of genistein and daidzein with CDs complexes were performed at lower temperatures (24–45 °C). Therefore, there is no information about isoflavones and CD phase solubility at high temperatures. Based on phase solubility diagrams found in the literature, it was observed that when performed at lower temperatures, increasing *α*- and *γ*-CD concentrations up to 10% in samples, the quantities of dissolved genistein increased. When using *β*-CDs, the results showed that genistein solubility increased. However, the results presented in the literature showed genistein solubility only with 2% concentration *β*-CD on a phase solubility diagram [22]. The solubility of daidzein with *β*-CDs also increased when the temperature was increased. Therefore, it is possible that daidzein complexes are formed more intensively at temperatures of up to 40 °C. In the literature, the formation of the genistein complex with cyclodextrins decreases with the increasing temperature [35,36].

### 3.3. Effect of 50% Ethanolic and CD-Assisted Extractions on the Aglycone Yield

In order to increase the isoflavone yield in extract, it was decided to use organic solvent as a co-solvent with CDs, because aglycones are compounds, which are more hydrophobic than hydrophilic; therefore, their solubility in water is limited. After the complex with CDs degrades, a precipitate of isoflavones can form in the water. Using organic solvent can prevent this process.

In the literature, other organic solvents were studied in relation to cyclodextrins, and their role as a co-solvent was observed. In the study of H. Yoshii et al., it was determined that only ethanol, methanol, acetonitrile, isopropyl alcohol and n-propyl alcohol can act as solvents and cannot fully compete with the drug for inclusion [37]. Conventional solid–liquid extraction commonly uses hydroalcoholic mixtures, and an increasing popularity of ethanol can be noticed in the literature due to its food-grade and less toxic nature [38]. L. Y. Yoshiara et al. optimized soy isoflavone extraction with different solvents and reported that using pure organic solvents for isoflavone extraction was not efficient, suggesting that the use of these extraction solvents in binary or ternary mixtures with water could be more advantageous [39]. In this study, 50% ethanol was selected as a safe solvent to use, so that the extracted isoflavones could later be used in nutraceutical production. Additionally, these results were supported by results presented in Table 4.

**Figure 6:**
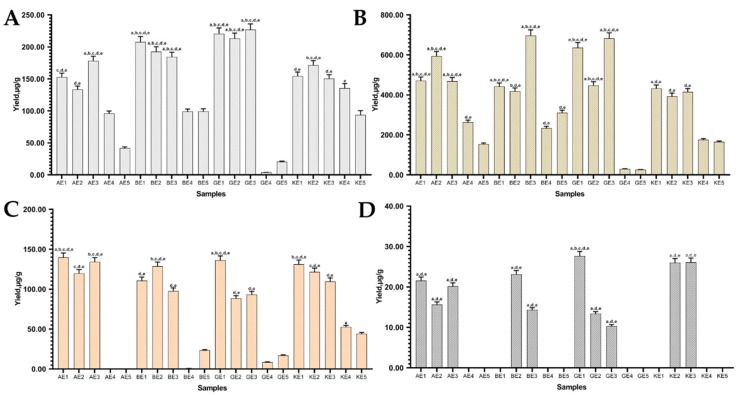
Quantitative yield of isoflavone aglycones ((**A**) genistein and (**B**) daidzein) and glycosides ((**C**) genistin and (**D**) daidzin) using excipients (1%) and 50% ethanol as a solvent. Control samples without excipients (KE1-5), samples with *α*-CD (AE1-5), *β*-CD (BE1-5), *γ*-CD (GE1-5). ^a^ *p* < 0.05 vs. KE1, ^b^ *p* < 0.05 vs. KE2, ^c^ *p* < 0.05 vs. KE3, ^d^ *p* < 0.05 vs. KE4, ^e^ *p* < 0.05 vs. KE5. Sample codes are provided in Table 1.

When using 50% of ethanol as a solvent, the highest yields of daidzein (*p* < 0.05) were detected in BE3 and GE3 samples using *β*-CDs and *γ*-CDs, respectively (Figure 6B). The highest yield of genistein was observed in sample GE3 (226.97 ± 9.08 µg/g) (Figure 6A), although similar results were obtained in samples GE1-2 and BE1. The genistein and daidzein contents in the BE3 sample were 184.30 ± 7.37 µg/g and 697.167± 27.88 µg/g; in the GE3 sample, they were 226.96 ± 9.07 µg/g and 682.90 ± 27.31 µg/g.

By changing the solvent from water (Figure 3) to 50% ethanol (Figure 6), the aglycone yield in the extract increases on average by 2–3 times. This was observed using all CDs, except *β*-CD, which reduced genistein yield by 1.17 times. Using *α*-CD in the extraction increased genistein yield by 3.32 times, and *γ*-CD increased the yield by 2.36 times. Daidzein amounts increased with all CDs—3.64, 2.31 and 3.06 times (*α*-, *β*- and *γ*-CD, respectively). As in the previous results, by using water as a solvent, the concentrations of isoflavones, obtained using only ultrasound without thermal hydrolysis, were low.

Boonyarattanakalin et al. studied the role of ethanol as a co-solvent in the cyclodextrin inclusion complexation. In the study, it was determined that ethanol has an effect on the formation of the cyclodextrin inclusion complex. It can act as a guest molecule, interfere with complex formation and compete with other guest molecules, but due to the different *α*-, *β*- and *γ*-CD cavity sizes, it interacts differently with each CD. Ethanol mainly forms complexes with *β*-CD [40]. Therefore, genistein and ethanol compete for *β*-CD. Due to the ethanol and CD interaction, the yield of isoflavones did not differ significantly compared with the control samples (KE1-5) as it did when CDs were used with purified water.

High yields of genistin and daidzin (Figure 6A,B) in the extracts showed that they were not fully hydrolyzed. The boiling point of 50% ethanol is lower than that of purified water. CDs form inclusion complexes more intensively at higher temperatures, so it is important to choose the right temperature [41]. Isoflavone aglycones can be unstable at high temperatures and break down [42]. The degradation reaction rate of isoflavones reaches its peak at ∼95 °C for genistein and at ∼98 °C for daidzein [43]. The boiling temperature of ethanol (50%) is 92 °C, and purified water has a boiling point of 100 °C. Therefore, the 8 °C difference can affect aglycones. Consequently, it can be assumed that glycosides are completely hydrolyzed to aglycones in purified water, using HRE or thermal hydrolysis, but may degrade due to exposure to high temperatures, yet some of them could be protected by the cyclodextrin–aglycone complex [17]. This is why using 50% ethanol results in more aglycones and their glycosides being recovered.

As mentioned before, not all the glycosides were hydrolyzed. The highest yields (*p* < 0.05) of genistin and daidzin were found in GE1 and AE1 samples (Figure 6A,B). Comparing the samples prepared with excipients, it was observed that when using *α*-CDs, the average genistin and daidzin amounts were higher than when using *β*-, *γ*-CD or no excipients in the extracts (control samples).

## 4. Conclusions

The results indicate that the use of *α*-, *β*- and *γ*-CDs during extractions increases the yield of isoflavones significantly. The use of purified water with CDs enhanced the isoflavone yields compared with samples without excipients.

The recovery of isoflavone aglycones from plant materials is influenced by the solvent, extraction time and temperature. Long extraction times and high temperatures increase the chance of the oxidation of CDs and the degradation of isoflavone aglycones, which decrease the yield of isoflavones in the extracts. To obtain a high amount of isoflavones, the water temperature should be closely monitored to avoid the degradation of compounds.

When using purified water as a solvent and increasing concentrations of CDs in the extraction media from 1 to 5%, daidzin yield increased on average by 1.06 (*α*-CDs), 1.4 (*β*-CDs) and 1.25 (*γ*-CDs) times. The amount of genistein increased using *α*- and *γ*-CDs (1.28 and 1.12 times *α*- and *γ*-CDs, respectively), but decreased using *β*-CD.

The highest genistein yield was detected in sample B1 258.67 ± 10.34 µg/g, which was prepared using *β*-CD and purified water. The highest daidzin yields were detected in BE3 and GE3–697.16 ± 27.88 and 682.90 ± 27.31 µg/g (using *β*- and *γ*-CD in 50% ethanol, respectively).

Our study demonstrated that the *α*-, *β*- and *γ*-CD-assisted extraction of *Trifolium pratense* L. enhanced the isoflavone yield compared to extraction using the same conditions without excipients. Increasing the levels of naturally occurring isoflavone aglycones from herbal materials can create new opportunities for the application of these bioactive compounds in food, nutraceutical and medical fields.

## Figures and Tables

**Table 1:** Extraction conditions used for the experiment.

Sample Code	Extraction Method ***	Hydrolysis Method	Time, min	Solvent *	Excipient	Excipient:Extract Ratio
KV1 **	HR	-	60	Purified water	-	-
KV2 **	UA	thermal	10			
KV3 **			30			
KV4 **		-	10			
KV5 **			30			
KE1 **	HR	-	60	50% Ethanol	-	-
KE2 **	UA	thermal	10			
KE3 **			30			
KE4 **		-	10			
KE5 **			30			
A1	HR	-	60	Purified water	*α*-CD	1:100
A2	UA	thermal	10			
A3			30			
A4		-	10			
A5			30			
AP1	HR	-	60			5:100
AP2	UA	thermal	10			
AP3			30			
AP4		-	10			
AP5			30			
AE1	HR	-	60	50% Ethanol		1:100
AE2	UA	thermal	10			
AE4			30			
AE5		-	10			
AE6			30			
B1	HR	-	60	Purified water	*β*-CD	1:100
B2	UA	thermal	10			
B3			30			
B4		-	10			
B5			30			
BP1	HR	-	60			5:100
BP2	UA	thermal	10			
BP3			30			
BP4		-	10			
BP5			30			
BE1	HR	-	60	50% Ethanol		1:100
BE2	UA	thermal	10			
BE3			30			
BE4		-	10			
BE5			30			
G1	HR	-	60	Purified water	*γ*-CD	1:100
G2	UA	thermal	10			
G3			30			
G4		-	10			
G5			30			
GP1	HR	-	60			5:100
GP2	UA	thermal	10			
GP3			30			
GP4		-	10			
GP5			30			
GE1	HR	-	60	50% Ethanol		1:100
GE2	UA	thermal	10			
GE3			30			
GE4			10			
GE5			30			

* Solvent and herbal material ratio is the same in all the samples—10:0.3, respectively. ** Control samples prepared without excipients. *** HR—heat-refluxed method; UA—ultrasound-assisted method.

**Table 2:** Recoveries (± RSD (%), *n* = 6) of daidzein, genistein, daidzin and genistin from red clover extracts.

Compound	± RSD *** (%), *n* = 6	
	UA *	UAH **
Daidzein	2.27	0.56
Genistein	3.10	3.61
Daidzin	0.82	0.65
Genistin	1.14	0.48

* Red clover sample sonicated for 10 min, at 40 °C; ** red clover sample sonicated for 10 min, at 40 °C and then heated under reflux for 1 h; *** recovery standard derivatization.

**Table 3:** Concentrations of isoflavones recovered from the samples with CDs.

Sample	Genistein, µg/mL	Daidzein, µg/mL	Genistin, µg/mL	Daidzin, µg/mL
Isoflavones without CDs	97.295	95.743	125.419	139.456
*α*-CDs prepared with isoflavones in purified water	96.701	95.487	125.271	139.222
*β*-CDs prepared with isoflavones in purified water	97.086	95.487	125.305	139.351
*γ*-CDs prepared with isoflavones in purified water	96.952	95.484	125.356	139.198
*α*-CDs prepared with isoflavones in 50% ethanol	97.053	95.487	125.386	139.382
*β*-CDs prepared with isoflavones in 50% ethanol	97.121	95.649	125.289	139.406
*γ*-CDs prepared with isoflavones in 50% ethanol	97.107	95.658	125.356	139.399

**Table 4:** Extraction conditions influence on yield of isoflavones.

Sample Code	Method	Solvent	Conditions	Daidzein, µg/mL	Genistein, µg/mL	Daidzin, µg/mL	Genistin, µg/mL
EU1(KE4)	UAE	50% EtOH	40 °C, 10 min	175.93	135.60	0.00	52.53
EU2(KE5)	UAE	50% EtOH	40 °C, 30 min	93.73	165.83	0.00	44.07
EU3	UAE	50% EtOH	60 °C, 10 min	98.89	121.21	0.00	41.22
EU4	UAE	50% EtOH	60 °C, 30 min	91.52	112.14	0.00	38.53
EU5	UAE	70% EtOH	40 °C, 10 min	56.15	69.02	0.00	79.33
EU6	UAE	70% EtOH	40 °C, 30 min	52.74	55.61	0.00	98.12
EU7	UAE	70% EtOH	60 °C, 10 min	84.12	106.35	0.00	84.62
EU8	UAE	70% EtOH	60 °C, 30 min	81.31	64.52	0.00	91.45
EU9	UAE	96% EtOH	40 °C, 10 min	21.22	147.33	0.00	89.24
EU10	UAE	96% EtOH	40 °C, 30 min	19.47	125.66	0.00	99.25
EU11	UAE	96% EtOH	60 °C, 10 min	73.54	119.41	0.00	92.17
EU12	UAE	96% EtOH	60 °C, 30 min	69.79	103.45	0.00	94.86
W1(KV4)	UAE	Purified water	40 °C, 10 min	51.06	1.23	0.00	0.00
W2(KV5)	UAE	Purified water	40 °C, 30 min	48.65	6.72	0.00	0.00
W3	UAE	Purified water	60 °C, 10 min	32.15	0.00	0.00	0.00
W4	UAE	Purified water	60 °C, 30 min	36.91	0.00	0.00	0.00

## Data Availability

Not applicable.
